# Comparing the effects of a mindfulness versus relaxation intervention on romantic relationship wellbeing

**DOI:** 10.1038/s41598-020-78919-6

**Published:** 2020-12-10

**Authors:** Johan C. Karremans, Gesa Kappen, Melanie Schellekens, Dominik Schoebi

**Affiliations:** 1 Behavioural Science Institute, Radboud University, Nijmegen, The Netherlands; 2 Helen Dowling Institute, Bilthoven, The Netherlands; 3 University of Fribourg, Fribourg, Switzerland

**Keywords:** Psychology, Human behaviour

## Abstract

There is increasing scientific interest in the potential association between mindfulness and romantic relationship wellbeing. To date, however, experimental studies using active control groups and testing dyadic effects (i.e. examining both actor and partner effects) are lacking. In the current study, romantically involved individuals engaged for 2 weeks daily in either guided mindfulness exercises, or guided relaxation exercises. Participants, and their partners, completed measures of relationship wellbeing at pre- and post-intervention, and at 1-month follow up. The mindfulness intervention significantly promoted relationship wellbeing, for both participants (i.e. actor effects) and their partners (i.e. partner effects). However, these findings did not significantly differ from changes in relationship wellbeing in the relaxation condition. Theoretical implications of these findings for understanding the association between mindfulness and romantic relationship wellbeing are discussed. Moreover, the findings are discussed in light of recent debates about the relative lack of proper control groups in mindfulness research.

## Introduction

The past decade has witnessed a steep increase in scientific research on mindfulness, revealing that mindfulness as a personality trait (i.e. trait mindfulness), and the practice and training of mindfulness, are associated with a range of beneficial outcomes for the individual. Mindfulness, which can be defined as the use of attention to bring awareness to current moment experiences in body and mind while maintaining a non-reactive and accepting attitude^1^, has been linked to reductions in stress, depressive symptoms, rumination, and negative affect, and increases in cognitive functioning, effective emotion regulation, and positive affect^2–4^. While some recent studies have described potential downsides of mindfulness^5–7^, in general mindfulness seems to hold considerable promise in alleviating distress and promoting individual wellbeing.

It has been suggested that the effects of mindfulness may generalize beyond the individual into the interpersonal domain^8,9^. More specifically, both in- and outside academia various claims have been made about the potential benefits of mindfulness in promoting romantic relationship functioning^10,11^. The theoretical basis for this general prediction includes several related lines of reasoning^12–14^. First, bringing non-reactive awareness to current moment experiences (including thoughts, emotions, and bodily sensations), and in particular to negative experiences, tends to facilitate the attenuation of those experiences^15,16^. In the context of a romantic relationship, during a heated discussion, or when one experiences negative feelings towards the partner, simply identifying and recognizing such feelings in a non-reactive way may reduce their intensity, as compared to actively resisting such experiences. Second, cultivating mindful awareness to experiences should promote accurate identification of feelings and thoughts that otherwise may go unnoticed, but nevertheless may automatically guide responses to the partner. For example, early-stage identification of potential relationship-threatening feelings or thoughts provides an opportunity to refrain from acting on them, and in addition, it is a prerequisite to communicate about those experiences and resolve tensions in the relationship in a constructive manner^17^. Third, and related, mindfulness should promote the early-stage recognition of experiences that are caused by stressors *outside* of the relationship (e.g. job stress; financial worries). There is ample evidence that such outside stressors can be a major source of relationship distress^18^. If attended to with non-reactive awareness, outside stressors should be less likely to have an automatic negative impact on the relationship, perhaps partly because partners are more likely to communicate about them. Finally, in addition to such relatively specific mechanisms, more general accounts for a potential association between mindfulness and romantic relationship functioning include that mindfulness has been linked to heightened empathy, compassion, and attachment security^19,20^, which are central factors in the functioning and wellbeing of a romantic relationship.

Despite these theoretical possibilities, research on mindfulness in romantic relationship functioning is still relatively scarce, and relies mostly on self-reported levels of trait mindfulness. Trait mindfulness, which represents between-person variation in the extent to which one is mindful across time and situations in daily life, has been associated with secure attachment^21,22^, lowered stress responses to relationship conflict^23,24^, more constructive conflict styles^25^, increased partner acceptance^26^, better sexual satisfaction^27,28^, and more generally, romantic relationship satisfaction^29–31^. There is, however, considerable debate regarding the validity of self-report measures of mindfulness^32^. Concerns include whether people can accurately assess awareness and attention lapses (particularly in retrospect), and there may be substantial response biases, for example depending on experience with mindfulness practice (e.g. guessing the ‘correct’ response^33^). Thus, while these findings on the association between trait mindfulness and romantic relationship outcomes are generally supportive of theoretical predictions, they should be considered in light of the limitations of self-report measures of trait mindfulness.

Moreover, correlational findings of trait mindfulness and indicators of relationship wellbeing do not address causality. A key question therefore is whether the training and cultivation of mindfulness can causally augment relationship wellbeing. Only a few studies have addressed this issue directly. In a randomized wait-list controlled study, Carson and colleagues examined the effects of participating in an 8-week mindfulness-based intervention program on a variety of relationship outcomes. Twenty-two couples in the intervention group, in which both partners participated, were compared with 22 couples in a waiting-list group^34^. Results indicated that compared to the wait-list the intervention was effective in promoting relationship satisfaction, connectedness, acceptance of each other, and reducing relationship distress (among other things). These results were maintained at 3 months follow-up. Notably, subsequent analyses of the data revealed that these couple improvements could be explained in terms of enhanced relationship excitement^35^. In other words, participating together in the intervention triggered feelings of excitement among partners, which enhanced relationship satisfaction (consistent with theory and research on the self-expansion model^36^). This raises the question whether the relationship enhancing effects in this study could be attributed to mindfulness training, or whether engaging in any other exciting activity together would have enhanced relationship satisfaction^37^. More recently, Khaddouma and colleagues published the results of an uncontrolled pilot study, showing that twenty participants who engaged in an 8-week standardized mindfulness program (mindfulness-based-stress-reduction; MBSR) significantly increased in relationship satisfaction from before to after the training; no such increases were found in their partners^38^. Finally, Schellekens and colleagues found that cancer patients and their partners, who took part in an MBSR program, showed significant decreases in psychological distress as compared to a waitlist group, but they found no significant increases in relationship satisfaction, neither among patients or their partners^39^. In addition to the small samples, an important limitation of these studies is that no (active) control condition was included, and it is therefore hard to tell whether any effects were due to mindfulness training per se.

Recently, Kappen and colleagues examined the effects of a low-dose mindfulness intervention on romantic relationship outcomes, as compared to a minimally active control condition^40^. In this study, one member of a couple engaged in a 12-day mindfulness intervention program. In addition to psycho-education on mindfulness, participants practiced mindfulness on a daily basis via guided instructions that were delivered through a website, and they reported briefly on their relationship experiences every night. In the control condition, participants received psycho-education, and also reported on their relationship experiences daily, but did not engage in mindfulness practice. In both conditions, relationship satisfaction and partner acceptance increased from pre- to post-intervention, but overall, there were no significant differences in change between the mindfulness intervention and control condition. Notably, among participants relatively low (versus high) in trait mindfulness at the start of the study, there was a stronger increase in relationship satisfaction in the intervention as compared to the control condition. While this study included a minimally active control group (i.e. participants in the control group also reported on their relationship daily, and received some psycho-education on mindfulness), control participants did not engage in any activity that replaced the mindfulness exercises, again making it difficult to infer whether effects were due to mindfulness practice in particular.

To the best of our knowledge, the studies discussed above are the only studies to date that sought to examine the causal influence of mindfulness training on romantic relationships, but all lacked or were limited in their use of an active control condition. This restriction relates to a recently expressed critique concerning the scientific literature on mindfulness more generally^32,41^. Studies that include proper active control groups are relatively rare in mindfulness research, limiting the conclusions that can be drawn about the effectivity and working mechanisms of mindfulness training.

## The current research

The goal of the present research was to examine the causal effects of a relatively short mindfulness intervention (2 weeks) on the well-being of romantic relationships. In other domains, such relatively short mindfulness interventions have been shown to be effective (e.g. regarding anxiety and depression^42^; individual happiness^43^; job satisfaction^44^). Importantly, we compared the mindfulness intervention with an active control intervention. As recommended by MacCoon and colleagues^45^, a proper active control condition should (1) match activities in the mindfulness intervention as closely as possible, (2) consist of activities that represent a plausible active ingredient in their own right (so as to match positive intervention expectations), with the only difference that (3) these ingredients do not involve mindfulness training. As an example, in a study using an active control group that fulfills these criteria, a relaxation intervention was compared to the effects of a mindfulness intervention on several outcomes of individual well-being (e.g., distress, rumination)^46^. It was found that mindfulness training benefitted participants, but not significantly more so than relaxation training (for similar results^47^). In a recent study, using a similar design, mindfulness training led to significantly greater self-reported resilience among firefighters as compared to relaxation training^48^.

In the current study, we used a similar approach, and compared the effects of engaging in mindfulness exercises daily for a period of 2 weeks with the effects of engaging in relaxation exercises. For the current article, we examined a number of relationship outcomes as indicators of overall relationship wellbeing, including relationship satisfaction and relationship distress as global evaluations of the relationship, and more specific outcomes, namely partner acceptance^26^, perceived connectedness, and relationship excitement.

One individual of a couple engaged in the intervention, and we examined the intervention effect on relationship outcomes as perceived by both the trainee (i.e. actor effects) *and* her or his partner (i.e. partner effects), who was not enrolled in the intervention. Only a few studies in this research area have examined such *dyadic* effects^29,49,50^. That is, most previous research has examined whether an individual’s level of mindfulness is associated with his or her own reports of relationship well-being. Romantic relationship partners, however, are fundamentally *interdependent*, meaning that changes in one partner affect outcomes of the other partner as well^51^. Examining such dyadic effects are essential to understand the impact of mindfulness in romantic relationship functioning and wellbeing.

## Methods

### Participants

The study was preregistered at the OSF. Throughout this method section, we indicate when and why we diverted from the preregistered plans. Participants were recruited via an independent Dutch research agency (http://www.flycatcher.eu), with a nation-wide panel of over ten thousand members. Panel members were invited via e-mail to fill in a first questionnaire assessing eligibility. Participants were required to be currently involved in a romantic relationship with a minimum duration of 1 year, living together with their partner and be over 18 years of age. A total of 1291 panel members filled in the eligibility questionnaire, of which 1233 qualified. Initially, we aimed to recruit at least 60 couples per intervention group, based on an a priori power analysis. Furthermore, based on available grant resources, we aimed to collect more than the minimum required 60 couples to be able to further explore the data. Due to an accidental mistake at the research agency, who performed the recruitment, many more couples were approached and included in the study (the research agency took care of the resulting extra costs of the study). The 1233 eligible panel members were randomly assigned to either the mindfulness intervention or relaxation intervention group, and were invited to fill in the informed consent and the first questionnaire of the study. At this point, partners of panel members were invited to join the study. In 80.2% of the couples (N = 989), the partners agreed to participate in the study. By the end of this procedure, there were 509 couples in the mindfulness intervention group, and 480 in the relaxation intervention group. Of the invited panel members, 55.9% returned all study measures, and 53.6% of partners returned all study measures, resulting in complete couple data for 562 dyads: n = 306 in the mindfulness intervention group, and n = 256 in the relaxation intervention group.

Participants and their partners were between 21 and 83 years old (*M* = 49), and had been in a relationship for 23 years on average. About 96% were in a heterosexual relationship, 3% in a homosexual relationship, and 0.7% indicated ‘other.’ About 75% of the couples were married, and 72% had children. About 62% of the participants who engaged in the intervention exercises were female. Couples received € 22.50 in exchange for their participation, and couples with complete data were included in a raffle for 10 vouchers of € 25. All participants provided written informed consent. The study was approved by the Ethics Committee Faculty of Social Sciences (EC2015-0903-304), Radboud University, and was conducted in accordance with relevant regulations and guidelines.

In the remainder of the article, we refer to panel members who took part in the mindfulness or control intervention (i.e. who engaged in the intervention exercises) as *intervention participants,* and refer to their partners as *partners.*

### Procedure

#### Intervention participants

For intervention participants, the study consisted of four elements: (1) pre-intervention baseline assessment in the week before the training period, (2) 2 weeks of mindfulness/relaxation intervention on working days, that included a short daily questionnaire, (3) post-intervention assessment in the week after the intervention period, (4) follow-up assessment 1 month after the training had finished. Links to the questionnaires and intervention materials were sent via email.

### Partners

For partners, the study consisted of three elements that took place parallel to the assessment times of the panel members: (1) pre-measure baseline assessment in the week before their partner’s intervention period, (2) a post-measure assessment in the week after their partner’s intervention period, (3) follow-up measures 1 month after the participant’s intervention had finished. Distribution of the questionnaires was similar to that for intervention participants.

### Intervention

#### Mindfulness

Mindfulness instructions consisted of daily 10-min, audio-guided exercises, recorded by the third author, who is a certified mindfulness trainer. Exercises consisted of the following components: (1) practical instructions about posture (e.g. “Sitting on a chair for this exercise, feet on the ground, back straight, hands resting in the lap.”), (2) short grounding in present moment experiences by paying attention to posture and the breath (e.g. “focusing attention on breathing."), (3) directing of attention towards experiences (thoughts, emotions, and bodily sensation; e.g. “What is in awareness right now, are there any thoughts?”), (4) Instruction to view experiences as transient events in the mind (a *decentered* attitude; e.g. “Without getting immersed in the content of thought, noticing what kind of thought there is at the moment—thoughts that come and go.”), (5) instruction to carry this quality of awareness into interpersonal interactions with others and especially the romantic partner (e.g. “In situations where you are in contact with your partner, becoming aware of thoughts and feelings that are present, and what you can notice in your body. Viewing these experiences as transient events, as we have practiced.”). To illustrate how to adopt a mindful attitude toward personal experiences, the instructions included different metaphors that are often used in mindfulness training, emphasizing the transient nature of experiences (e.g. experiences are like an ongoing stream of water in a waterfall, or like clouds moving along a blue sky). Over the course of the 2 weeks, participants listened to three different versions of this exercise, differing slightly in wording.

#### Relaxation

Similar to the mindfulness instructions, relaxation instructions consisted of a 10-min, audio-guided exercise, also recorded by the third author. Relaxation exercises consisted of the following components: (1) Practical instructions about posture. (2) Guidance to successively tense and relax muscles of different body parts. (3) Instruction to carry any resulting feeling of relaxation into interpersonal interactions with others and especially with the romantic partner. Over the course of the 2 weeks, participants were offered three versions of this exercise, differing slightly in wording. A transcript of the instructions and audio files for both conditions can be found at the OSF.

### Measures

An overview of all variables assessed per moment of measurement can be found at the OSF. In the preregistration, additional and more detailed hypotheses were formulated, that are not tested in the current article (e.g. moderation by stress; stress spillover effect, i.e. is the association between external daily stress and relationship outcomes will be weakened after mindfulness training). For the current article, to examine the broader research question whether mindfulness as compared to relaxation intervention promotes romantic relationship wellbeing, we focused on a number of related and complementary relationship outcome variables—relationship satisfaction, relationship distress, connectedness, partner acceptance, and relationship excitement. As noted in the introduction, relationship satisfaction and distress can be regarded as more global evaluations of the relationship, while connectedness, partner acceptance, and relationship excitement are more specific relationship outcomes. These variables were previously assessed in the few existing studies that examined causal effects of mindfulness intervention, but that did not include active control conditions (as discussed in the introduction^34,38,39,41^). Moreover, these relationship variables have previously been associated with trait mindfulness^25,26,52,53^.

Both intervention participants and their partners completed the same relationship measures, at all three timepoints, except for partner acceptance (which will be explained below).

### Pre-, post- and follow-up measures

#### Global relationship satisfaction

At all three timepoints, global relationship satisfaction was assessed with the Relationship Assessment Scale (RAS)^54^, consisting of seven items (e.g. “How well does your partner meet your needs?”). Answers were given on a scale from 1 = *low* to 7 = *high*. Across the three timepoints, α’s > 0.90 for both the intervention participants and for partners.

#### Single-item relationship satisfaction

In addition to the RAS as an indicator of global relationship satisfaction, on all three timepoints participants indicated on a single-item scale how satisfied they currently were with their relationship, when evaluating the past 2 weeks (i.e. “How satisfied were you with your relationship during the past 2 weeks?”). Answers were given on a scale from 1 = *unsatisfied* to 7 = *very satisfied*.

#### Relationship distress

At all three timepoints, the level of relationship distress was assessed with one item: "During the past 2 weeks, how many problems, stress or conflict have you experienced in your relationship?", answers were given on a scale from 1 = *none at all* to 7 = *a lot*.

#### Perceived connectedness

At all three timepoints, perceived connectedness to the partner was measured with three items (e.g. “During the last 2 weeks, when I was in contact with my partner, I felt really connected to my partner.”), from 1 = *completely disagree* to 7 = *completely agree*. For both the intervention participants and for partners, α’s > 0.90 at all three time points.

#### Partner acceptance

At all three timepoints, for intervention participants, partner acceptance was measured with the 5-item Partner Acceptance Scale^26^ [PAS; e.g. “My partner does not have to be the perfect partner,” “Frankly, I would like my partner to be the ideal partner” (reverse coded)], from 1 = *completely disagree* to 7 = *completely agree*. For all three timepoints, α’s > 0.70. In partners, we measured *perceived* partner acceptance, in which items were reframed to assess the extent to which the partner felt accepted by the other (i.e. by the intervention participant; e.g. “My partner does not need me to be the perfect partner,”). There was some variation in reliability; pre-measure α = 0.61; post-measure α = 0.69; follow-up measure α = 0.71.

#### Relationship excitement

At all three timepoints, both partners indicated their perceived level of relationship excitement on one item (i.e. “How exciting is your relationship at the moment?”) from 1 = *not exciting at all* to 7 = *very exciting*.

*Adherence.* On each day, participants indicated their level of adherence to the intervention instructions (i.e. “How seriously have you followed the instructions for the exercise today?”, from 1 = *not seriously at all* to 7 = *very seriously*).

As can be read in the OSF overview document, the study included additional outcome measures (e.g. rejection anxiety, accommodation, pre-intervention relationship commitment). As noted, in the current article, we used the relationship evaluation measures as described to answer the more general and previously unanswered question whether mindfulness as compared to relaxation can promote relationship wellbeing. Together, the results of these related and complementary measures should provide a valid answer to this central research question. As can be seen in the preregistration, we also mentioned a number of control variables that could potentially affect the results (e.g. personality characteristics, demographics). For ease of interpretation and clarity, we report uncontrolled models here, but provide the results of a controlled model as supplementary material (of which the results were very similar). Notably, in the preregistration we also mentioned that the more specific measures (i.e. acceptance, connectedness, excitement) that we used here as indicators of relationship wellbeing may statistically mediate the effect of mindfulness (versus relaxation) intervention on the more global measures (i.e. relationship satisfaction, distress). We did not further perform and report these statistical tests for mediation. We consider this beyond the scope of the current general research question, and importantly, as can be read below, we found little support for significant differences between interventions regarding changes in relationship satisfaction or distress.

## Results

The current data feature multiple sources of nonindependence. Repeated measurements at the pre- post- and follow-up time point were correlated within individuals, and the participant and partners’ reports on their relationship outcomes were correlated within couples. Furthermore, the study examined multiple outcome measures that were correlated within individuals and across dyads. Because ignoring these sources of (co)variation might bias significance tests^55,56^, we used a model that incorporates correlated residuals among repeated measurements and dependent variables, both within individuals and between participants and their partners. To this end, we tested aa multilevel model with a dyadic structure, in which we modelled participants’ and their partners’ dependent variables as separate equations with covariances among the residuals^57^. Furthermore, we modelled the repeated measures as clustered within dyads. In each equation, we estimated a random intercept, and random slopes for pre-to-post and pre-to-follow-up time contrasts. Random intercepts and slopes across individuals and couples were then regressed on intervention group membership.

This statistical approach differed from the proposed analyses in the preregistration insofar as a series of separate, independent analyses were merged into a single model, allowing to account for patterns of contingencies between the different variables studied.

### Adherence

Analysis of the average scores of the daily adherence question across the 2 weeks indicated that the mindfulness and relaxation intervention groups did not differ on self-reported adherence, respectively *M* = 5.59, *SD* = 1.07, vs. *M* = 5.71, *SD* = 1.15, *F* (1, 561) = 1.676, *p* = 0.196*,* indicating that both groups took the exercises seriously to a similar degree.

### Premeasure differences

We first tested whether participants and their partners differed with respect to relationship outcomes within and between the two intervention groups (see Table 1). Participants who received the mindfulness intervention did not differ significantly from participants who received the relaxation intervention on any of the relationship outcomes pre-intervention. Also, comparisons between participants and their partners suggest no significant differences in relationship satisfaction or connectedness pre-intervention. However, pre-intervention, participants in the relaxation group reported more relationship distress than their partner, participants in both intervention groups reported more partner acceptance than their partner, and participants in the mindfulness group reported less relationship excitement than their partner. As can be seen in Tables 2 and 3, across the intervention groups, couples were on average relatively happy prior to the intervention (i.e., high in relationship satisfaction, *M*’s > 5.73, *SD*’s 0.96–1.03, on the 7-point scale, and relatively low in relationship distress, *M*’s < 2.68, *SD*’s 1.41–1.44, on the 7-point scale).

**Table 1:** Mean comparisons of pre-intervention outcomes for participants and partners for the two intervention groups.

	Comparison between intervention type Mindfulness vs. relaxation		Comparison within intervention type^a^ Participants vs. partners	
	Participants Mean difference (SE)	Partners Mean difference (SE)	Mindfulness Mean difference (SE)	Relaxation Mean difference (SE)
**Relationship outcomes**				
Global satisfaction	0.002 (0.075)	− 0.072 (0.063)	− 0.045 (0.044)	− 0.054 (0.051)
Single-item satisfaction	0.019 (0.079)	0.010 (0.076)	− 0.090 (0.064)	− 0.011 (0.062)
Relationship distress	0.053 (0.091)	0.074 (0.091)	0.040 (0.075)	**0.171** (0.077)
Connectedness	− 0.003 (0.069)	0.000 (0.071)	− 0.089 (0.057)	− 0.066 (0.060)
Partner acceptance	0.047 (0.067)	0.064 (0.065)	**0.134** (0.059)	**0.174** (0.064)
Relationship excitement	0.119 (0.097)	0.143 (0.094)	− 0**.261** (0.090)	− 0.142 (0.094)

Bold print indicates significant effects (p < 0.05); Participants mindfulness intervention N = 304, Participants relaxation intervention N = 254. ^a^Paired differences.

**Table 2:** Pre-post and pre-follow-up change in individual and relationship outcomes for *intervention participants* by intervention type.

	Intervention participants																Comparison	
	Mindfulness intervention (n = 304)								Relaxation intervention (n = 254)								Mindfulness intervention vs. Relaxation intervention	
	M	SD	B pre-post	SE	p	B pre-fu	SE	p	M	SD	B pre-post	SE	p	B pre-fu	SE	p	B/SE p-value	B/SE p-value
**Relationship outcomes**																		
Global satisfaction	**5.733**	1.067	**0.140**	0.023	0.000	**0.179**	0.027	0.000	**5.731**	1.114	**0.114**	0.034	0.001	**0.166**	0.035	0.000	− 0.634	− 0.295
																	0.524	0.777
Single-item satisfaction	**5.814**	1.304	**0.098**	0.047	0.038	**0.181**	0.052	0.000	**5.795**	1.351	0.096	0.048	0.050	**0.189**	0.057	0.001	− 0.029	0.104
																	0*.*979	0.920
Relationship distress	**2.687**	1.493	− **0.244**	0.065	0.000	− **0.236**	0.067	0.000	**2.634**	1.541	− 0.077	0.066	0.239	− **0.206**	0.073	0.005	1.796	0.303
																	0.072	0.761
Connectedness	**5.687**	1.138	**0.183**	0.037	0.000	**0.179**	0.048	0.000	**5.690**	1.162	**0.146**	0.045	0.001	0.074	0.055	0.179	− 0.638	− 1.419
																	0.519	0.155
Partner acceptance	**4.981**	1.067	**0.359**	0.044	0.000	**0.399**	0.048	0.000	**4.934**	1.162	**0.484**	0.045	0.000	**0.489**	0.048	0.000	**1.984**	1.343
																	**0.046**	0.179
Relationship excitement	**3.883**	1.612	− 0.092	0.078	0.238	− 0.031	0.082	0.705	**3.764**	1.636	0.105	0.080	0.188	**0.223**	0.082	0.006	1.759	**2.209**
																	0.078	**0.027**

Bold print indicates significant effects (p < 0.05).

**Table 3:** Pre-post and pre-follow-up change in relationship outcomes for *partners* by intervention.

	Intervention participants																Comparison	
	Mindfulness intervention (n = 304)								Relaxation intervention (n = 254)								Mindfulness intervention vs. Relaxation intervention	
	M	SD	B pre-post	SE	p	B pre-fu	SE	p	M	SD	B pre-post	SE	p	B pre-fu	SE	p	B/SE p-value	B/SE p-value
**Relationship outcomes**																		
Global satisfaction	**5.777**	1.067	**0.144**	0.026	0.000	**0.155**	0.029	0.000	**5.849**	1.043	**0.090**	0.033	0.006	**0.093**	0.039	0.018	− 1.286	− 1.265
																	0*.*193	0.200
Single-item Satisfaction	**5.861**	1.256	0.096	0.052	0.065	**0.124**	0.052	0.016	**5.851**	1.280	0.088	0.050	0.081	0.103	0.064	0.110	− 0.111	− 0.253
																	0.911	0.795
Relationship distress	**2.638**	1.470	− **0.221**	0.068	0.001	− **0.317**	0.067	0.000	**2.564**	1.565	− **0.182**	0.074	0.013	− **0.266**	0.079	0.001	0.394	0.495
																	0.695	0.622
Connectedness	**5.805**	1.162	0.019	0.041	0.644	− 0.043	0.050	0.388	**5.804**	1.209	− 0.025	0.046	0.582	0.013	0.053	0.805	− 0.710	0.778
																	0.472	0.439
Partner acceptance	**4.901**	1.067	**0.219**	0.049	0.000	**0.234**	0.049	0.000	**4.837**	1.091	**0.204**	0.053	0.000	**0.332**	0.055	0.000	0.205	1.324
																	0.832	0.183
Relationship excitement	**4.119**	1.565	− 0**.161**	0.076	0.033	− **0.166**	0.082	0.043	**3.976**	1.588	0.020	0.084	0.818	**0.187**	0.084	0.027	1.602	**2.992**
																	0.111	**0.003**

Bold print indicates significant effects (p < 0.05).

### Change in relationship outcomes—intervention participants

Next, we tested whether post and follow-up measurements differed from pre-measurements, and importantly, whether these differences varied by intervention group. Table 2 shows the estimates for these tests for intervention participants. Participants who received the mindfulness intervention reported significantly higher relationship satisfaction on both the global and single-item measures, lower relationship distress, higher connectedness, higher partner acceptance at post-measurement than at the pre-measurement. These changes were maintained at the follow-up measurement. Notably, no significant change was observed for relationship excitement.

Participants who received the relaxation intervention also reported higher relationship satisfaction (on both measures) and higher partner acceptance at post and follow-up measurements than at pre-measurement. Significant changes from the pre-measurement—specifically lower relationship distress, higher single-item relationship satisfaction, and more excitement—occurred only at the follow-up measurement, and were not significant at the post-measurement. A significant increase in connectedness at the post-measurement, as compared to the pre-measurement, was not maintained at the follow-up measurement. Importantly, change estimates did not differ between intervention groups, except for the pre- to post-measurement increase in partner acceptance (*p* = 0.046), and the pre- to follow-up increase in relationship excitement (*p* = 0.027), which were significantly lower for participants who received the mindfulness intervention.

### Change in relationship outcomes—partners

In Table 3, the results for partners are reported. As can be seen in this table, for both the mindfulness and the relaxation condition, partners also showed a significant increase in global relationship satisfaction and partner acceptance, and a significant decrease in relationship distress from pre- to post measurement, and these changes were maintained at the follow-up measurement. There were no significant changes in connectedness, and significant increases in the single-item relationship satisfaction report occurred only from the pre- to the follow-up measurement in the mindfulness intervention group. Interestingly, relationship excitement decreased from the pre- to post-measurement in the mindfulness group, and these decreases were still present at the follow-up measurement. In contrast, no significant change in relationship excitement occurred from pre-to post measurement in the relaxation group, but a significant increase from the pre- measurement was present at the follow-up measurement. Again, for most outcome variables, changes did not differ significantly between the two intervention groups. The only exception was relationship excitement, for which changes between pre- and follow-up measurements differed significantly between the intervention groups (*p* = 0.003).

In the supplementary materials, we report additional exploratory analyses. First, we report the results of a number of individual functioning measures that were included in the study, and that may account for changes in relationship outcomes. Second, we explored whether self-reported treatment adherence impacted these results. A detailed description of the results can be found in the online supplementary materials.

## General discussion

Does mindfulness promote the wellbeing of romantic relationships? The possible causal effect of mindfulness training on romantic relationships has received very little empirical attention so far. After 2 weeks of daily guided mindfulness practice, participants in the mindfulness intervention group reported significantly higher levels of relationship satisfaction, lower relationship distress, felt more connected to the partner, and were more accepting towards the partner (but not more excited about their relationship). These effects were maintained 1 month after the intervention. Their partners, who did not engage in mindfulness practice, also reported higher relationship satisfaction, less distress, and felt more accepted by their partners (but not more connected; and they reported actually less relationship excitement, a finding we further discuss below). In general, these findings may be considered as promising for the effectiveness of mindfulness intervention in promoting relationship wellbeing. A similar pattern of findings, however, was found in the active control condition. Participants who received a daily guided relaxation intervention for 2 weeks showed similar relationship benefits (in fact, they showed significantly more positive change in partner acceptance, and positive change in relationship excitement), as did their partners. Thus, while the current findings suggest that the daily practice of mindfulness generally can lead to various beneficial relationship outcomes, relaxation practice on a daily basis yielded comparable outcomes.

What is an adequate interpretation for these findings? First, these results might simply mean that any intervention, similar in structure, would positively affect self-reports of relationship wellbeing (sometimes referred to as a *trial effect*^58^). For example, the intervention may have prompted participants to reflect on their relationship, which could have promoted positive feelings and thoughts about the relationship (although in theory it might also do the opposite, of course). Also, engaging in an intervention could have led participants to report desired rather than actual outcomes. The fact that intervention participants showed similar changes in both conditions, across most relationship outcome measures, and that their partners showed similar changes (except regarding relationship excitement), makes this a possible interpretation of the current findings.

An alternative interpretation might be that the relationship benefits may be genuine, both in the mindfulness as well as relaxation intervention. In the introduction, we discussed several theoretical reasons why mindfulness may promote relationship wellbeing (for more details^10^), and the results in the mindfulness group may reflect such theorized effects. Similarly, the results obtained in the relaxation group may reflect true relationship benefits of daily relaxation exercises. For example, daily relaxation might reduce overall psychological and physiological stress levels, which in turn might positively affect how people behave and respond to their partners^18,59^. Accordingly, both mindfulness and relaxation intervention may have promoted relationship wellbeing, but possibly through different mechanisms. The obtained effects in both groups, however, also may have been caused by a similar ‘relaxation mechanism.’ While mindfulness is theoretically distinguishable from relaxation, in reality it is possible that the mindfulness exercises in our study increased feelings of relaxation to a similar extent as the relaxation exercises. As noted previously^60^, the historical development of (mindfulness) meditation and relaxation techniques as therapeutic strategies in the past century show substantial overlap, and indeed one of the challenges of studying the effects of mindfulness is to distinguish them from ‘mere’ relaxation effects^61^.

It could be theorized that longer mindfulness training is required to promote relationship wellbeing above and beyond effects of relaxation. Participants practiced mindfulness for 2-weeks, engaging in a relatively short guided mindfulness meditation each day. While such relatively short mindfulness interventions have revealed significant effects on various outcome measures^42–44^ (but rarely have been compared with active control groups), effects of mindfulness practice may occur and generalize to real life outcomes only after more extensive training, when paying non-judgmental attention becomes a more or less automatic manner of relating to one’s experiences^62,63^. How people cope with and respond to their experiences is the result of a lifelong process, and therefore difficult to ‘re-program’^64,65^. Likewise, particularly in long-term romantic relationships, interaction patterns between partners and appraisals about the relationship tend to become habitual, and are therefore difficult to change in the short run^66^. Thus, what the ‘dosage’ of mindfulness practice should be to potentially promote relationship wellbeing, and how much for whom, requires more research.

The results regarding relationship excitement revealed a somewhat different pattern than the other relationship outcome measures. There was no significant change in relationship excitement in the mindfulness group among intervention participants. This finding seems inconsistent with previous findings by Carson and colleagues, who found increases in relationship excitement after a mindfulness intervention^35^. The present results may suggest that it was not mindfulness per se leading to such changes in their study, but the fact that both partners engaged in their intervention may have explained an increase in relationship excitement. Interestingly, in the current study the partners of the mindfulness intervention participants actually reported significant *declines* in relationship excitement. We have previously speculated that there may be theoretical reasons to predict such declines^13^. For example, mindfulness may be associated with reductions in impulsivity, a potentially important source of relationship excitement^67^. This finding awaits future research and replication.

The results of the current study speak to the broader issue of the need for proper active control conditions in mindfulness research. A substantial percentage of research on mindfulness is lacking active control conditions, using waiting list controls, or examining changes in a variable of interest from pre- and post-mindfulness intervention^68,69^. Such studies can be informative to see whether mindfulness intervention is associated with any changes on variables of interest, or for example to study moderators of any effects of mindfulness intervention (e.g. for whom it does and does not ‘work’). Although the inclusion of an active control condition made the current findings perhaps more ambiguous and difficult to interpret, it highlights the importance of studying whether changes associated with mindfulness intervention can be attributed uniquely to mindfulness. In many previous articles on mindfulness research, the conclusion that mindfulness positively affects a certain outcome of interest is often unwarranted when an active control group is lacking^32,40^. The current findings raised a number of issues (as discussed above) that may remain unaddressed when control conditions are lacking, underscoring the need for active control groups to get a more nuanced and more comprehensive understanding of mindfulness and its effects, both for the individual and the relationship.

Similarly, the current findings have implications for couple intervention research more broadly. In the past decades, researchers have examined effects of various prevention and intervention couple programs. Some programs have obtained significant relationship benefits in the short- and longer-term^70,71^, others obtained negative outcomes (e.g. increased awareness that one is lacking relationship skills^72^, but often waiting list control groups are used to compare intervention outcomes. Relatively few studies used comparisons with active control groups, and if they did, similar effects have been found between target and control intervention groups^73^. Thus, there is a need for proper control interventions in order to examine relationship interventions more rigorously.

Before closing, some limitations should be noted. First, all outcome measures were self-reports, and future studies should include more objective measures of relationship functioning and wellbeing, such as observational coding of partner interactions, and/or physiological assessments of (relationship) distress. Second, as noted already, the interventions were relatively short. For example, examining the effects of longer protocolized interventions^65^, such as the mindfulness-based stress-reduction program on relationship outcomes, while including an active control intervention, would be a logical and important next step. Similarly, examining associations between length and frequency of mindfulness practice and relationship outcomes would be a valuable and complementary approach. Finally, a potential limitation of the current study is that the intervention in the current research targeted one member of the couple. While it is a theoretically interesting question whether the cultivation of mindfulness in one individual transfers to the relationship partner, mindfulness intervention might be more effective when both partners engage in the intervention.

The present study was the first to examine the *causal* impact of mindfulness training on romantic relationship outcomes using an active control group and testing both actor and partner effects, thereby extending previous research that has linked romantic relationship wellbeing mainly to self-report measures of mindfulness. The current study does not give definitive answers to the question whether or not mindfulness can causally affect relationship wellbeing, but does provide a compelling example of why research on mindfulness interventions would benefit from a wider use of active control groups, hopefully offering a springboard for future research.

## Supplementary Information

Supplementary Information.

## Data Availability

The data are available from the corresponding author on request, and will be made publicly available.
