# Supplementary material for: Comparing the effects of a mindfulness versus relaxation intervention on romantic relationship wellbeing
Source: Sci Rep. 2020 Dec 10;10:21696. doi: 10.1038/s41598-020-78919-6 (PMC7730385; doi:10.1038/s41598-020-78919-6)
Supplement: Supplementary file 1 — Supplementary Information. [file 41598_2020_78919_MOESM1_ESM.docx]

**Comparing the effects of a mindfulness versus relaxation intervention on romantic relationship wellbeing**

Online supporting materials

Johan C. Karremans*

Gesa Kappen

*Behavioural Science Institute, Radboud University, the Netherlands*

Melanie Schellekens

*Helen Dowling Institute, the Netherlands*

Dominik Schoebi

*University of Fribourg, Switserland*

*Corresponding author, [j.karremans@psych.ru.nl](mailto:j.karremans@psych.ru.nl)

**Online supporting materials**

**Additional analyses: Controlled model (I), Individual functioning (II), and treatment adherence (III)**

**Controlled model (I)**

In Tables 2alt and 3alt are the results, respectively for the intervention participants and partners, of the controlled model with covariates added as listed in the preregistration. Outcome measures were global relationship satisfaction, single-item relationship satisfaction, relationship distress, connectedness, partner acceptance, and relationship excitement. The predictors in the model were, in addition to intervention group: age, sex, relationship duration, trait mindfulness, insecure attachment, self-esteem, life satisfaction, extraversion, conscientiousness, openness to experience, and the partner’s relationship commitment, insecure attachment, and openness to experience. Other covariates that were mentioned in the preregistration, but that were found not to be significantly related to any of the outcome variables in preparatory analyses, were not included in this model (specifically: number of children, neuroticism, agreeableness, as well the partner’s age, sex, trait mindfulness, self-esteem, life satisfaction, neuroticism, agreeableness, extraversion, and conscientiousness). As can be seen when comparing the tables of the uncontrolled (in the article) and the controlled models (below), there were only slight changes in the results. For example, whereas the single-item satisfaction measure from pre- to post-intervention showed a significant increase in intervention participants in the uncontrolled model, *p* = .038, this change was not significant in the controlled model, *p* = .065. Importantly, the comparisons between intervention groups regarding any changes from pre-measurement to post- or follow-up measurement did not differ between the uncontrolled or controlled model. The conclusions based on this model do not change.

Table 1: Pre-post and pre-follow-up change in individual and relationship outcomes for *intervention participants* by intervention type

|  | Intervention participants | | | | | | | | | | | | | | | | | | | | Comparison | |  |
| --- | --- | --- | --- | --- | --- | --- | --- | --- | --- | --- | --- | --- | --- | --- | --- | --- | --- | --- | --- | --- | --- | --- | --- |
|  |  | Mindfulness intervention (n= 304) | | | | | | | | | Relaxation intervention (n=254) | | | | | | | | | Mindfulness intervention vs. Relaxation intervention | | | |
|  | *M* | | *SD* | *B pre-post* | *SE* | *p* | *B pre-follow-up* | *SE* | *p* | *M* | | *SD* | *B pre-post* | *SE* | *p* | *B pre-follow-up* | *SE* | *p* | *B/SE* | | | *B/SE* |  |
| *Relationship outcomes* |  | |  |  |  |  |  |  |  |  | |  |  |  |  |  |  |  |  | | |  |  |
| Global Satisfaction | **5.733** | | 1.067 | **.130** | .023 | .000 | **0.176** | 0.027 | .000 | **5.728** | | 1.115 | **.113** | .034 | .001 | **.174** | .035 | .000 | -.433  *p*=.665 | | | - .054  *p*=.957 |  |
| Single-item Satisfac. | **5.812** | | 1.304 | .087 | .047 | .065 | **0.186** | 0.051 | .000 | **5.795** | | 1.346 | **0.100** | .049 | .043 | **.208** | .056 | .000 | .192  *p*=.848 | | | .301  *p*=.764 |  |
| Relationship Distress | **2.692** | | 1.493 | **-.241** | .065 | .000 | **-0.234** | 0.067 | .000 | **2.627** | | 1.533 | -.085 | .066 | .201 | **-.211** | .073 | .004 | 1.718  *p*=.086 | | | .239  *p*=.811 |  |
| Connectedness | **5.683** | | 1.138 | **.172** | .037 | .000 | **0.177** | 0.047 | .000 | **5.692** | | 1.160 | **.156** | .044 | .001 | .086 | .055 | .118 | -.281  *p*=.779 | | | -1.264  *p*=.206 |  |
| Partner acceptance | **4.993** | | 1.067 | **.343** | .043 | .000 | **0.395** | 0.047 | .000 | **4.922** | | 1.155 | **.477** | .045 | .000 | **.489** | .049 | .000 | **2.140**  ***p*=.032** | | | 1.399  *p*=.162 |  |
| Relationship Excitement | **3.872** | | 1.612 | -.099 | .077 | .198 | -0.022 | 0.08 | .786 | **3.774** | | 1.631 | .096 | .081 | .235 | **.241** | .081 | .003 | 1.725  *p*=.084 | | | **2.292**  ***p*=.022** |  |

Note. bold print indicates significant effects (p<.05). Coefficients were adjusted for participant age, sex, relationship duration, pre-intervention mindfulness, insecure attachment, self-esteem, life satisfaction, extraversion, conscienciousness, openness to experience, and partner insecure attachment, commitment, and openness to experience.

Table 2: Pre-post and pre-follow-up change in relationship outcomes for *partners* by intervention

|  | Intervention participants | | | | | | | | | | | | | | | | | | Comparison | |
| --- | --- | --- | --- | --- | --- | --- | --- | --- | --- | --- | --- | --- | --- | --- | --- | --- | --- | --- | --- | --- |
|  |  | Mindfulness intervention (n= 304) | | | | | | | | Relaxation intervention (n=254) | | | | | | | | Mindfulness intervention vs. Relaxation intervention | | |
|  | | *M* | *SD* | *B pre-post* | *SE* | *p* | *B pre-fu* | *SE* | *p* | *M* | *SD* | *B pre-post* | *SE* | *p* | *B pre-fu* | *SE* | *p* | *B/SE* | | *B/SE* |
| *Relationship outcomes* | |  |  |  |  |  |  |  |  |  |  |  |  |  |  |  |  |  | |  |
| Global Satisfaction | | 5.782 | 1.067 | **.136** | .026 | .000 | **.157** | .030 | .000 | 5.843 | 1.043 | **.085** | .033 | .013 | **.092** | .039 | .019 | -1.234  *p*=.217 | | -1.359  *p*=.174 |
| Single-item Satis. | | 5.862 | 1.256 | .092 | .052 | .075 | **.137** | .053 | .009 | 5.848 | 1.280 | .087 | .050 | .092 | .109 | .064 | .086 | - .072  *p*=.942 | | - .347  *p*=.729 |
| Relationship Distress | | 2.644 | 1.470 | **-.196** | .069 | .004 | **-.320** | .068 | .000 | 2.557 | 1.565 | **-.169** | .074 | .021 | **-.274** | .079 | .000 | .275  *p*=.783 | | .454  *p*=.650 |
| Connectedness | | 5.809 | 1.162 | .010 | .042 | .802 | -.036 | .050 | .475 | 5.798 | 1.209 | -.020 | .046 | .669 | .027 | .053 | .598 | - .498  *p*=.619 | | .897  *p*=.370 |
| Partner acceptance | | 4.913 | 1.067 | **.205** | .049 | .000 | **.227** | .048 | .000 | 4.824 | 1.091 | **.210** | .053 | .000 | **.343** | .055 | .000 | .063  *p*=.950 | | 1.565  *p*=.118 |
| Relationship Excitement | | 4.115 | 1.565 | **-.170** | .074 | .022 | **-.175** | .080 | .028 | 3.974 | 1.588 | .022 | .084 | .789 | **.186** | .084 | .029 | 1.717  *p*=.086 | | **3.106**  ***p*=.002** |

Note. bold print indicates significant effects (p<.05). Coefficients were adjusted for participant age, sex, relationship duration, pre-intervention mindfulness, insecure attachment, self-esteem, life satisfaction, extraversion, conscienciousness, openness to experience, and partner insecure attachment, commitment, and openness to experience.

**Additional analyses II: Individual functioning**

The current study also included several indicators of individual functioning, and we explored whether any relationship effects of the intervention could be explained by improvements in individual functioning, in particular increased levels of decentering (i.e. the ability to distance oneself from experiences, seeing them as fluctuating mental events^1^), self-control, positive affect, and reductions in rumination. These individual processes have been associated with mindfulness training in previous research,^2,3,4^ and have been identified previously as potential mechanisms of how mindfulness may promote relationship wellbeing.^5^

***Individual outcomes: pre-, post- and follow-up measures***

*Decentering.* Decentering was assessed with five items capturing the extent to which people disengaged from thoughts and feelings, e.g. “During the last two weeks I struggled with letting go off my thoughts” (reversed), “Over the last two weeks, I saw thoughts and feelings as transient,” α’s ranging from .65 to .75. We constructed this measure for the purpose of this study based on previous measures of decentering.^6^

*Rumination*. A single item, “In the last two weeks, when I experienced distress, I ruminated a lot” measured self-reported levels of rumination.

*Affect.* A single item, "Over the past two weeks, I felt... " (1 *= not at all well* to 7 *= very well*) measured affect, from negative to positive.

*Self-control*. To measure self-control, the 4-item inhibition subscale of the Deficits in Executive Functions Scale was used.^7^ Sample items were: In the past two weeks, e.g. “I was unable to inhibit my reactions or response toward events or others,” “I acted without thinking,” all α’s > .81.

***Results***

We applied the same statistical strategy for the individual outcomes as for the relationship outcomes. Using multilevel modelling, with the repeated measures as clustered within intervention participants, we simultaneously tested the individual outcome measures, incorporating correlated residuals between the measures. As can be seen in the table below (Table 3), intervention participants in both intervention groups showed an increase in decentering and positive affect, and a decrease of self-control deficits and rumination, from pre- to post-measurement, and maintained this change at the follow-up measurement. Changes did not differ significantly between the intervention groups, except for decreases in rumination: participants in the mindfulness intervention maintained stronger decreases from the pre- to follow-up measurement than participants in the relaxation intervention (*p*=.028). This latter finding is consistent with previous findings by Jain and colleagues^8^, who found that there were significantly stronger decreases in rumination after a mindfulness versus relaxation intervention.

Table 3: Pre-post and pre-follow-up change in individual outcomes for *intervention participants* by intervention

|  | Intervention participants | | | | | | | | | | | |
| --- | --- | --- | --- | --- | --- | --- | --- | --- | --- | --- | --- | --- |
|  | Mindfulness intervention (n= 306) | | | | | Relaxation intervention (n=256) | | | | | Comparison  Mindfulness intervention vs. Relaxation intervention | |
|  | Pre  *M (SD)* | Post  *M (SD)* | Follow-up  *M (SD)* | *B (SE)* | | Pre  *M (SD)* | Post  *M (SD)* | Follow-up  *M (SD)* | *B (SE)* | | *B (SE)* | |
|  |  |  |  | Pre-Post | Pre-FU |  |  |  | Pre-Post | Pre-FU | Pre-Post | Pre-FU |
| Decentering | 3.87 (.91) | 4.56 (.98) | 4.62 (.98) | **.64 (.05)** | **.69 (.05)** | 3.93 (.91) | 4.55 (1.02) | 4.54 (1.10) | **.63 (.05)** | **.61 (.06)** | .01 (.07) | .08 (.08) |
| Positive affect | 5.12 (1.27) | 5.36 (1.22) | 5.45 (1.18) | **.30 (.06)** | **.39 (.07)** | 5.06 (1.20) | 5.40 (1.03) | 5.40 (1.14) | **.34 (.06)** | **.34 (.07)** | -.04 (.09) | .05 (.09) |
| SC^*^ deficits | 3.11 (1.18) | 2.81 (1.07) | 2.73 (1.09) | **-.41 (.06)** | **-.49 (.06)** | 3.22 (1.23) | 2.70 (1.08) | 2.63 (1.13) | **-.52 (.06)** | **-.59 (.06)** | .11 (.08) | .10 (.08) |
| Rumination | 4.60 (1.60) | 3.26 (1.62) | 3.28 (1.58) | **-1.09 (.08)** | **-1.06 (.08)** | 4.34 (1.64) | 3.39 (1.62) | 3.56 (1.70) | **-.96 (.08)** | **-.78 (.10)** | -.13 (.12) | **-.28 (.13)** |

Note. bold print indicates significant effects (p<.05); SC = self-control

To explore potential mechanisms underlying the intervention effects for relationship outcomes, we examined whether pre-post change in individual functioning was predictive of concurrent (pre-post) or prospective (pre-follow up) change in relationship outcomes. To this end, we extended our models by including pre-post latent change scores of individual functioning as predictors of the change estimates in relationship functioning, while adjusting for main effects of individual functioning change on relationship outcomes.

The results of a model that included individual functioning predictors simultaneously did not yield any significant results (all *p* > .246). Next, we tested change scores of each individual functioning predictor separately. A model testing latent change in affect as predictors of change in relationship outcomes did not suggest significant associations (*p* > .074). Testing change in rumination as predictor of change in relationship outcomes suggested that pre-to-post intervention reductions in rumination were associated with improvement or maintenance in perceived connectedness at the follow-up time point *(b= -1.947, p= .000)*, but change in rumination did not predict change in any other relationship outcome (*p* > .241). Next, examining changes in decentering as predictor of change in relationship outcomes suggested that increases in decentering predicted improved relationship satisfaction on the long term (pre-to-follow-up increments; *b= 1.129, p= .012*), but otherwise yielded no significant results (*p* > .104). Finally, examining change in self-control deficits as predictor of change in relationship outcomes suggested no meaningful associations between these change scores (*p* > .154). Overall, these analyses suggested no meaningful association between the magnitude of change in individual functioning and the change in relationship functioning, and they thus provided no support for changes in individual functioning to operate as a mediator for intervention effects on relationship outcomes.

**Additional analyses II: Treatment adherence**

In additional analyses, we examined the potential relevance of self-reported treatment adherence for the observed effects. To this end, we included the average of the treatment adherence ratings, reported daily during the treatment phase. We tested main effects to examine possible correlations between participants’ treatment adherence and their individual or relationship outcomes, interaction effects with time to examine whether treatment adherence was related to the magnitude of change from the pre- to the post or follow-up measurement, and a three-way interaction with time and intervention group to examine whether treatment adherence was differentially related to change in individual or relationship functioning for the two intervention groups.

The analyses yielded inconsistent results. Treatment adherence was not significantly related to participants’ relationship satisfaction, distress reports or acceptance. It was significantly related to participants’ higher overall levels of connection (*b* = .127; *p*=.005). It was not differentially related to relationship functioning of participants in the two intervention groups.

In terms of individual functioning, participants’ reported treatment adherence was not related to affect, rumination or decentering, or self-control deficits. Treatment adherence was not related differentially to individual functioning between the two groups, except for associations with self-control deficits, where the mindfulness group featured a negative association with self-control deficits, and the relaxation group no association (*b=* -.134, *p*= .039).

For partners, treatment adherence was significantly associated with higher relationship satisfaction (*b=* .096, *p*= .017), lower relationship distress (*b=* -.167 *p*= .005), and stronger connection (*b=* .097 *p*= .041). It was not significantly related to partners’ acceptance levels, and associations between treatment adherence and relationship functioning did not differ between intervention groups.

Regarding intervention effects, we found no consistent evidence for treatment adherence predicting changes in relationship functioning or individual functioning. Treatment adherence only predicted reductions in self-control deficits from pre- to post measurement (*b=* -.123 *p*= .010). Treatment adherence differentially predicted change in partners’ relationship distress in the two intervention groups (*b=* -.176 *p*= .026), suggesting a stronger reduction of relationship distress from pre- to post measurement in the mindfulness group, as compared to the relaxation group, at high treatment adherence. Similarly, participants’ pre- to follow-up changes in relationship satisfaction were more positive in the mindfulness group than in the relaxation group, at high levels of treatment adherence (*b=* .076 *p*= .019). Participants’ change in self-control deficits, however, were more positive at high levels of treatment adherence for the mindfulness group, as compared to the relaxation group (*b=* .166 *p*= .015).

References

1. Bernstein, A. et al. Decentering and related constructs: A critical review and metacognitive processes model. *Perspect. Psychol. Sci.* **10**, 599-617 (2015).
2. Bowlin, S. L., & Baer, R. A. Relationships between mindfulness, self-control, and psychological functioning. *Pers. Individ. Differ.* **52,** 411-415 (2012).
3. Malinowski, P., & Lim, H. J. Mindfulness at work: Positive affect, hope, and optimism mediate the relationship between dispositional mindfulness, work engagement, and well-being. *Mindfulness* **6,** 1250-1262 (2015).
4. Raes, F., & Williams, J. M. G. The relationship between mindfulness and uncontrollability of ruminative thinking. *Mindfulness* **1,** 199-203 (2010).
5. Karremans, J. C., Schellekens, M. P., & Kappen, G. Bridging the sciences of mindfulness and romantic relationships: A theoretical model and research agenda. *Pers. Soc. Psychol. Rev.* **21,** 29-49 (2017).
6. Hadash, Y., Lichtash, Y., & Bernstein, A. Measuring decentering and related constructs: Capacity and limitations of extant assessment scales. *Mindfulness***8,** 1674-1688 (2017).
7. Barkley, R. A. *Barkley Adult ADHD Rating Scale-IV (BAARS-IV)* (Guilford Press, 2011).
8. Jain, S. et al. A randomized controlled trial of mindfulness meditation versus relaxation training: effects on distress, positive states of mind, rumination, and distraction. *Ann. Behav. Med.* **33,** 11-21 (2007).
